# 14-3-3ε Facilitates Replication of Multiple Viruses Accompanied by Reduced NF-κB Pathway Activity in Largemouth Bass (*Micropterus salmoides*)

**DOI:** 10.3390/ani16213368

**Published:** 2026-10-25

**Authors:** Bingbing Sun, Tianlai Hong, Kexin Hu, Huifang Zeng, Meisheng Yi, Kuntong Jia

**Affiliations:** 1 School of Marine Sciences, Sun Yat-sen University, Guangzhou 510275, China; 2 Guangdong Provincial Key Laboratory of Marine Resources and Coastal Engineering, Guangzhou 510006, China; 3 Southern Marine Science and Engineering Guangdong Laboratory (Zhuhai), Zhuhai 510275, China

**Keywords:** Ms14-3-3ε, VHSV, LMBV, RGNNV, NF-κB

## Abstract

Frequent outbreaks of viral diseases and the increasing prevalence of polyviral co-infections have severely constrained the sustainable development of largemouth bass (*Micropterus salmoides*) aquaculture. Identifying conserved host factors exploited by diverse viruses is critical for developing broad-spectrum antiviral strategies, yet the key host molecules underlying cross-viral infection susceptibility in teleosts remain poorly characterized. Here, this research identifies *Micropterus salmoides* 14-3-3ε protein (Ms14-3-3ε) as a critical pro-viral host factor that facilitates the replication of multiple phylogenetically distinct viruses by negatively modulating NF-κB-mediated inflammatory responses. *Ms14-3-3ε* was ubiquitously expressed in healthy largemouth bass tissues and was significantly upregulated both in vivo and in vitro upon infection with viral hemorrhagic septicemia virus (VHSV), largemouth bass virus (LMBV), and red-spotted grouper nervous necrosis virus (RGNNV). Functional gain- and loss-of-function assays demonstrated that *Ms14-3-3ε* serves as a broad-spectrum pro-viral factor: its overexpression markedly enhanced the transcription and protein expression of core viral genes, whereas *Ms14-3-3ε* knockdown effectively suppressed the transcription expression of all three viruses and protein levels of VHSV G and RGNNV CP. Mechanistically, Ms14-3-3ε negatively regulates the NF-κB signaling pathway in a dose-dependent manner by specifically inhibiting the phosphorylation of p65 at the Ser536 residue. This suppression further downregulates the expression of downstream pro-inflammatory effector genes NLRP3 and IL-1β, thereby blunting host innate immune defense responses to multi-viral proliferation. Collectively, the results demonstrate that Ms14-3-3ε facilitates the replication of taxonomically distinct fish RNA and DNA viruses associated with reduced NF-κB pathway activity in largemouth bass. This study provides a novel, conserved host target for the development of broad-spectrum antiviral strategies in aquaculture.

## Simple Summary

Recurrent viral outbreaks and escalating polyviral co-infections severely constrain sustainable aquaculture, particularly for largemouth bass (*Micropterus salmoides*). Identifying conserved host factors co-opted by multiple viruses is critical for the development of broad-spectrum antiviral strategies. This study identifies Ms14-3-3ε as a key broad-spectrum pro-viral host factor in largemouth bass. *Ms14-3-3ε* is markedly upregulated upon infection with RNA viruses (VHSV and RGNNV) and a DNA virus (LMBV). Functional assays demonstrate that *Ms14-3-3ε* promotes replication of these taxonomically distinct pathogens. Mechanistically, Ms14-3-3ε suppresses p65 phosphorylation at Ser536 in a dose-dependent manner, which attenuates NF-κB signaling and downregulates downstream pro-inflammatory genes NLRP3 and IL-1β, thereby dampening host innate immune defenses. This finding establishes a conserved host target for broad-spectrum antiviral intervention in aquaculture.

## 1. Introduction

Aquaculture serves as a strategic industry safeguarding food security and ecological sustainability. Nonetheless, frequent disease outbreaks have severely hampered the healthy development of economically important aquatic species. Epidemiological analyses indicate that co-infection with multiple pathogens has become prevalent in aquatic animals [1], which may alter host susceptibility and reshape host–pathogen interaction dynamics, and modify the pathological features of infection [2]. For instance, penaeid shrimp are frequently co-infected with white spot syndrome virus (WSSV) and infectious hypodermal and hematopoietic necrosis virus [3], while salmonids are prone to concurrent infection with infectious hematopoietic necrosis virus and infectious pancreatic necrosis virus [4]. Such combined infections exacerbate pathological lesions, complicate disease progression and markedly elevate mortality rates. Concurrently, they drive dysregulation of host immune responses and drastically reduce the efficacy of conventional single-target vaccines and antiviral therapies [5]. Accordingly, identifying conserved host factors that modulate infection progression across multiple viruses is essential for elucidating the molecular mechanisms of virus–host interactions, as well as for advancing broad-spectrum disease-resistant breeding and developing novel antiviral targets.

The largemouth bass (*Micropterus salmoides*) constitutes a globally significant freshwater aquaculture species that is susceptible to multiple highly pathogenic viruses. Among these, viral hemorrhagic septicemia virus (VHSV), largemouth bass virus (LMBV), and red-spotted grouper nervous necrosis virus (RGNNV) have attracted extensive attention due to their severe economic impacts. Specifically, VHSV is a negative-sense single-stranded RNA virus that causes systemic septicemia, with a mortality rate exceeding 90% [6]. As a double-stranded DNA virus, LMBV predominantly targets visceral organs such as the liver, spleen, and kidney, manifesting as surface hemorrhages, ulcerative dermatitis, exophthalmia, and visceral necrosis [7]. RGNNV is a positive-sense single-stranded RNA virus that primarily attacks the central nervous system of fish, causing characteristic neurological symptoms such as spiral swimming and swim bladder hyperinflation [8]. Despite profound differences in genome types, replication cycles, and pathogenic mechanisms, all three viruses can infect and propagate in largemouth bass. Co-infection involving these viruses has been reported; for example, LMBV and a rhabdovirus were simultaneously isolated from diseased largemouth bass [9], and co-infection can cause more severe mortality than single infection. During diagnostic screening of diseased fish from farms, some of these viruses were occasionally detected simultaneously, although these field observations are not presented as published epidemiological data. These observations suggest that largemouth bass may possess conserved host factors and signaling pathways that are convergently exploited by distinct viruses. Identifying such potential broad-spectrum host factors is important for understanding heterologous multi-virus co-infections and for uncovering universal antiviral targets in teleosts.

The 14-3-3 proteins constitute a family of highly conserved acidic dimeric proteins (existing as either homo- or heterodimers) widely distributed throughout eukaryotes [10]. Seven isoforms, namely β/α, ε, γ, η, σ, ζ/δ and θ (also known as τ), have been identified in mammals. They specifically recognize phosphorylated serine/threonine motifs (e.g., RSXpSXP and RX(Y/F)XpSXP) on target proteins and orchestrate a diverse array of physiological and pathological processes, including cell cycle regulation, apoptosis, signal transduction, cytoskeleton rearrangement and innate immunity [11,12]. Accumulating evidence has demonstrated that the 14-3-3 family plays a pivotal role in core pathways of host antiviral innate immunity, namely the retinoic acid-inducible gene I-like receptor (RLR) and nuclear factor κB (NF-κB) signaling pathways. Specifically, mammalian 14-3-3ε acts as a mitochondrion-targeted chaperone that stabilizes the interaction between RIG-I and TRIM25, thereby promoting K63-linked ubiquitination and mitochondrial translocation of RIG-I to trigger MAVS-dependent antiviral signaling [13]. Meanwhile, 14-3-3η promotes MDA5 oligomerization and intracellular redistribution in a dose-dependent manner [14]. Intriguingly, the NS3 protein of Zika virus (ZIKV) can block host antiviral innate immune responses by competitively binding to both 14-3-3ε and 14-3-3η [15]. Within the NF-κB pathway, 14-3-3θ selectively downregulates TLR2/MyD88-dependent NF-κB activation whilst enhancing the TLR4/IRF3-dependent antiviral response, thereby modulating the production of pro-inflammatory cytokines IL-6, IL-8 and TNF-α in different ways [16]. Additionally, 14-3-3 proteins can negatively regulate NF-κB activation by directly inhibiting IKKβ to maintain immune homeostasis [17], a physiological “brake” frequently exploited for viral immune evasion. For instance, hepatitis B virus (HBV) polymerase and human immunodeficiency virus type 1 (HIV-1) Vpr proteins suppress NF-κB signaling to promote viral replication [18,19]. Collectively, this unique “positive chaperone–negative brake” dual-regulatory mechanism within the RLR and NF-κB pathways renders the 14-3-3 family a core hub for balancing host antiviral immunity and inflammation, making them prime targets for diverse viruses.

Compared with mammals, the fish 14-3-3 protein family exhibits significant isoform expansion and functional differentiation due to evolutionary duplication events. Whole-genome comparative analysis reveals that non-teleost fish possess only 5-7 14-3-3 genes, whereas teleosts have expanded this repertoire to 9–11 genes as a result of the teleost-specific genome duplication (TGD/3R-WGD), with the number reaching 17–25 in certain polyploid groups like salmonids [20,21]. Although preliminary progress has been made in elucidating the role of the fish 14-3-3 family in innate immunity, current research has predominantly focused on the β and ζ isoforms. For instance, crucian carp 14-3-3β/α-A interacts with the glycoprotein of spring viremia of carp virus, participating in the host immune response against viral infection [22]. In zebrafish, 14-3-3β/α-A has been identified as a maternally derived peptidoglycan-binding protein that protects early embryos from pathogenic bacterial challenges [23]. Notably, although 14-3-3ε serves as a core regulatory node of the RLR and NF-κB pathways in mammals, it has only been cloned and characterized in a limited number of fish. Whether and how 14-3-3ε modulates antiviral innate immune signaling in fish remains largely elusive.

In this study, the functional role and regulatory mechanism of largemouth bass 14-3-3ε during infection with VHSV, LMBV, and RGNNV were investigated. The open reading frame (ORF) of Ms14-3-3ε was cloned and characterized. Subsequent functional assays confirmed that Ms14-3-3ε promotes infection by VHSV, LMBV, and RGNNV, accompanied by reduced NF-κB pathway activity. The present findings provide valuable theoretical insights and potential molecular targets for clarifying the role of 14-3-3 proteins in teleosts, and for developing broad-spectrum antiviral strategies against VHSV, LMBV, and RGNNV.

## 2. Materials and Methods

### 2.1. Fish, Cells, Virus, and Reagents

Largemouth bass fry (body length: 6–8 cm and mean body weight 4.0 g) were purchased from Taihui Aquatic Fry Wholesale Company (Guangzhou, China). The fish were acclimated in laboratory aquaria for two weeks, maintaining a water temperature of 25 °C in aerated water. The fish were fed commercial pellets twice per day, and 1/3 of the water in the tanks was replaced. During acclimation, fish were monitored for health and absence of injury and confirmed virus-negative by qRT-PCR before viral infection assays. All experimental procedures involving largemouth bass were strictly approved by the Ethics Committee of Sun Yat-sen University and conducted in accordance with relevant guidelines. Prior to tissue collection, fish were euthanized by immersion in an overdose of MS-222 (Sigma-Aldrich, St. Louis, MO, USA), followed by rapid tissue collection to minimize suffering. During the experiment, fish were monitored daily, and moribund or severely injured individuals were humanely euthanized according to predefined endpoints.

Largemouth bass muscle (MSM) cells were cultured at 28 °C in DMEM medium (Gibco, Thermo Fisher Scientific, Waltham, MA, USA) supplemented with 15% FBS (Techno, Tecnoblen S.A., Las Piedras, Uruguay).

VHSV-LB2018 (Genotype IVa) strain was isolated from a wild largemouth bass and propagated in fathead minnow (FHM) cells [24]. RGNNV was initially isolated from diseased juvenile sea perch in Guangdong Province, China, and propagated in LJB cells [25]. LMBV was derived from diseased largemouth bass collected in Guangdong Province, China, in 2025 [26]. All viral stocks were stored at −80 °C until use.

Anti-Flag (Cat. No. M20008L), anti-Myc (Cat. No. M20002L), anti-β-actin (Cat. No. M2011L), IL-1β (Cat. No. PS18840) and NLRP3 antibodies (Cat. No. P60622R3) were purchased from Abmart (Shanghai, China). Polyclonal anti-G serum was produced by immunizing rabbits with prokaryotically expressed VHSV G protein and provided by Shanghai Kebai Biotechnology (Shanghai, China). HRP-conjugated anti-rabbit IgG and anti-mouse IgG secondary antibodies were obtained from Beyotime (Shanghai, China). Rabbit monoclonal antibodies against NF-κB p65 (D14E12, Cat. No. 8242T) and Phospho-NF-κB p65 (Ser536) (93H1, Cat. No. 3033T) were acquired from Cell Signaling Technology (Danvers, MA, USA). Anti-p65 and anti-phospho-p65 (Ser536) were raised against synthetic peptides corresponding to residues surrounding Glu498 and Ser536 of human NF-κB p65/RelA, respectively. The Ser536 designation refers to the position homologous to human p65 Ser536. Anti-RGNNV CP polyclonal antibody was custom-generated by KEBIOCHEM company (Shanghai, China) against RGNNV CP. It detected a single band at ~40 kDa corresponding to RGNNV CP.

### 2.2. RNA Extraction and cDNA Synthesis

Total RNA was isolated from largemouth bass tissues and MSM cells using the FastPure Complex Tissue/Cell Total RNA Isolation Kit (R113-01, Vazyme Biotech Co., Ltd., Nanjing, China) according to the manufacturer’s protocols. Lysates were centrifuged at 12,000 rpm for 3–5 min, and the resultant supernatants were collected and mixed thoroughly with 0.5 volumes of absolute ethanol. The mixture was transferred to a FastPure RNA Column VI and centrifuged at 12,000 rpm for 30 s. The columns were then washed sequentially with Buffer RWA and Buffer RWB, respectively, and the RNA was finally eluted with RNase-free ddH_2_O. RNA concentration and quality were evaluated utilizing a NanoDrop 2000 spectrophotometer (Thermo Fisher Scientific, Waltham, MA, USA). For cDNA synthesis, the HiScript^®^ 1st Strand cDNA Synthesis Kit (+gDNA wiper) (Vazyme Biotech Co., Ltd., Nanjing, China) was employed. For qRT-PCR, cDNA was synthesized using Oligo(dT)_20_VN or random primers. For gene amplification, cDNA was synthesized using a mixture of Oligo(dT)_20_VN and Random hexamers. The reaction conditions were maintained at 37 °C for 45 min (to yield PCR products) or 15 min (to yield qRT-PCR products), and terminated at 85 °C for 5 s. The obtained cDNA was preserved at −20 °C for subsequent experiments.

### 2.3. Plasmid Construction

The cDNA sequence of *Ms14-3-3ε* was retrieved from the NCBI database (Accession No. XM_038708054.1). Specific primers (Ms14-3-3ε-3′-F and Ms14-3-3ε-5′-R) were designed based on this sequence to amplify the target ORF fragment via PCR. The PCR products were ligated into the eukaryotic expression vectors pCMV-(DYKDDDDK)-N and pCMV-Myc-N. The ligation mixtures were transformed into *Escherichia coli* Trans5α competent cells (TransGen Biotech Co., Ltd., Beijing, China). Positive clones were selected and verified by DNA sequencing. The reporter plasmids pRL-TK and pNF-κB-Luc were preserved in our laboratory. All primer sequences used in this study are detailed in Supplementary Table S1. Cell transfection was carried out using Lipofectamine™ 8000 (Beyotime, Shanghai, China) following the manufacturer’s protocol and previous reports.

### 2.4. Bioinformatics Analysis

The amino acid sequence of Ms14-3-3ε was predicted utilizing the NCBI ORF Finder tool (https://www.ncbi.nlm.nih.gov/orffinder/) (accessed on 5 June 2026). Genomic and protein sequences of 14-3-3ε orthologs were retrieved from the NCBI database. Multiple protein sequence alignments were performed using the MegAlign program embedded in the LASERGENE software package (DNASTAR 11.1). Subsequently, a phylogenetic tree was constructed with MEGA 6.0 via the neighbor-joining method based on the *p*-distance model. The bootstrap method with 1000 replicates was used to assess the reliability of each phylogenetic node.

### 2.5. In Vivo and in Vitro Viral Challenges

For the in vivo challenge assay, a total of 120 largemouth bass fry were used. For each treatment group (PBS, VHSV, LMBV, and RGNNV), 30 fish were randomly allocated into three tanks (10 fish per tank), and the three tanks served as three independent biological replicates. Fish were intraperitoneally injected with 200 μL of VHSV (1 × 10^7^ TCID_50_/mL), 200 μL of LMBV (2.1 × 10^8^ TCID_50_/mL), 200 μL of RGNNV (3 × 10^7^ TCID_50_/mL), or an equal volume of PBS (as the control group). At each time point (0, 6, 12, 24, 48, and 72 hpi), one fish was sampled from each tank, and each sampled brain was processed individually; the tank was the unit of statistical analysis (*n* = 3).

For the in vitro viral challenge, MSM cells were seeded in 6-well plates; the culture medium was removed prior to viral infection, and cells were inoculated with virus at the indicated multiplicities of infection (MOI = 1 for VHSV and RGNNV; MOI = 0.1 for LMBV, corresponding to the titers stated) and incubated at 28 °C for 2 h for virus adsorption, with gentle rocking every 15 min. The inoculum was then removed, cells were washed three times with PBS, and fresh medium was added. Cells and supernatants were harvested at the indicated time points. Cell samples were collected at 0, 6, 12, 24, 48, and 72 hpi for RNA isolation. Furthermore, MSM cells were subjected to Ms14-3-3ε overexpression or knockdown prior to viral infection or PBS treatment. Cells were harvested at 12 and 24 h post-infection (hpi), or at 24 hpi only for the assays indicated in the corresponding figures and figure legends. Total RNA extracted from all samples was used for subsequent quantitative real-time PCR (qRT-PCR) analysis.

### 2.6. TCID_50_ Assay

To assess progeny virus production, MSM cells were transfected with pCMV-Flag or pCMV-Flag-Ms14-3-3ε for 24 h and then infected with the indicated virus. Supernatants were collected at the indicated time points, clarified by centrifugation at 12,000× *g* for 10 min at 4 °C, and stored at −80 °C until titration. Viral titers were determined by the 50% tissue culture infectious dose (TCID_50_) assay. Confluent monolayers of MSM cells in 96-well plates were inoculated with 100 μL of 10-fold serial dilutions of the clarified supernatant (10^−1^ to 10^−10^), with 8 replicate wells per dilution, and incubated at 28 °C for 7 days. Infection was scored by the presence of cytopathic effect (CPE) under an inverted microscope. TCID_50_ values were calculated by the Spearman-Karber method and expressed as TCID_50_/mL. For statistical analysis, titers were log_10_-transformed prior to comparison.

### 2.7. RNA Interference

Three independent siRNAs targeting distinct regions of Ms14-3-3ε were designed and chemically synthesized by Tsingke company (Guangzhou, China). To minimize sequence-specific off-target effects, the three siRNAs were used as an equimolar pool at a final total concentration of 50 nM, such that each individual siRNA was present at ~16.7 nM, well below the concentration typically associated with prominent off-target silencing. The selection criterion was a significant reduction in Ms14-3-3ε mRNA. The siRNA sequences are listed in Supplementary Table S1. MSM cells were plated into culture dishes and incubated until reaching the appropriate confluence. Subsequently, cell transfection was performed with 50 nM of specific siRNA or a negative control siRNA (NC) using TSnanofect V2 transfection reagent (Tsingke Biotechnology Co., Ltd., Guangzhou, China), following the manufacturer’s protocols. Cells were harvested 24 h post-transfection, and the expression levels of genes involved in the NF-κB signaling pathway were further detected and analyzed.

### 2.8. Quantitative Real-Time PCR (qRT-PCR)

Total RNA was extracted and reverse-transcribed into cDNA as described above. qRT-PCR was carried out on a LightCycler 480 II real-time PCR system (Roche Diagnostics, Basel, Switzerland) using SYBR Green Master Mix (TaKaRa Bio Inc., Dalian, China). Specific primers for qRT-PCR were designed with Primer Premier 6.0 software (Supplementary Table S1). The qRT-PCR mixture consisted of cDNA template (2 μL), nuclease-free water (2.5 μL), SYBR Green PCR master mix (5 μL), and 0.25 μL of each forward and reverse primer, with a final primer concentration of 0.2 μM per primer. The amplification program was set as follows: initial denaturation at 95 °C for 10 min, followed by 40 cycles of 95 °C for 15 s and 60 °C for 30 s. No-template controls and no-reverse-transcriptase controls were included in each experimental run to exclude reagent contamination and genomic DNA interference, respectively. Melting curve analysis was performed post-amplification to verify amplicon specificity. Amplification efficiencies for all primer pairs were determined by standard curves generated from 5-fold serial dilutions of pooled cDNA. All primer pairs exhibited amplification efficiencies ranging from 92% to 104% with linear correlation coefficients (R^2^) > 0.99, meeting the requirements for relative quantification. *Msβ-actin* was selected as the reference gene. Its expression stability was validated across all examined tissues and under all viral infection conditions. Relative mRNA expression levels were calculated using the 2^−ΔΔCt^ method. All experiments were performed in three independent biological replicates.

### 2.9. Western Blotting

Pre-cultured MSM cells were transfected with the indicated plasmids using Lipofectamine™ 8000 (Beyotime, Shanghai, China). At 24 h or 48 h post-transfection, cells were rinsed with PBS and lysed in lysis buffer (20 mM Tris, pH 7.5; 150 mM NaCl; 1% Triton X-100; supplemented with sodium pyrophosphate, β-glycerophosphate, EDTA, Na_3_VO_4_, leupeptin and PMSF). Cell lysates were sonicated on ice using a JY92-IIDN ultrasonic cell disruptor (Ningbo Xinzhi Biological Technology Co., Ltd., Ningbo, China) at 300 W, with three cycles of 3 s sonication and 5 s intervals. Afterwards, the lysates were centrifuged at 16,000× *g* for 15 min at 4 °C. The resulting supernatants were mixed with SDS loading buffer for immunoblot analysis.

Protein samples were separated via 10% SDS-PAGE and then transferred onto 0.22 μm polyvinylidene difluoride (PVDF) membranes (Bio-Rad Laboratories, Hercules, CA, USA). The membranes were blocked with 5% non-fat dry milk diluted in TBST buffer (25 mM Tris-HCl, 150 mM NaCl, 0.1% Tween-20, pH 7.5) for 1 h at room temperature (RT). Subsequently, the membranes were incubated with appropriate primary antibodies overnight at 4 °C. After washing three times with TBST, the membranes were incubated with secondary antibodies for 1 h at RT. Immunoreactive protein bands were detected using ECL chemiluminescence reagents (Merck Millipore, Burlington, MA, USA) and visualized with a Minichemi 420 imaging system (Sage Creation, Beijing, China).

### 2.10. Dual-Luciferase Reporter Assay

Dual-luciferase reporter assays were conducted with the Dual-Luciferase Reporter Assay System (Promega, Madison, WI, USA) according to a previously established protocol [27]. MSM cells seeded in 24-well plates were co-transfected with the pNF-κB-Luc reporter plasmid, pCMV-Flag/Myc-Ms14-3-3ε, and 25 ng of the pRL-TK plasmid as an internal control. At 24 h post-transfection, cells were lysed using passive lysis buffer (Promega, Madison, WI, USA) for 15 min at RT. Luciferase activities were subsequently measured using a GloMax 20/20 Luminometer (Promega, Madison, WI, USA) in accordance with the manufacturer’s instructions. All experiments were performed in triplicate across three independent trials.

### 2.11. Statistical Analysis

All quantitative data were analyzed using GraphPad Prism 8.0 (GraphPad Software, San Diego, CA, USA). Two-tailed Student’s *t*-test was used for comparisons between two groups. Time-course and dose–response data were analyzed by one-way ANOVA followed by Dunnett’s multiple-comparison test versus the control group. Experiments combining treatment and time or virus and dose were analyzed by two-way ANOVA. Data are presented as mean ± SD; *n* = 3 independent biological replicates; *, *p* < 0.05; **, *p* < 0.01; ***, *p* < 0.001.

## 3. Results

### 3.1. Sequence Analysis of Ms14-3-3ε

**Figure 1:**
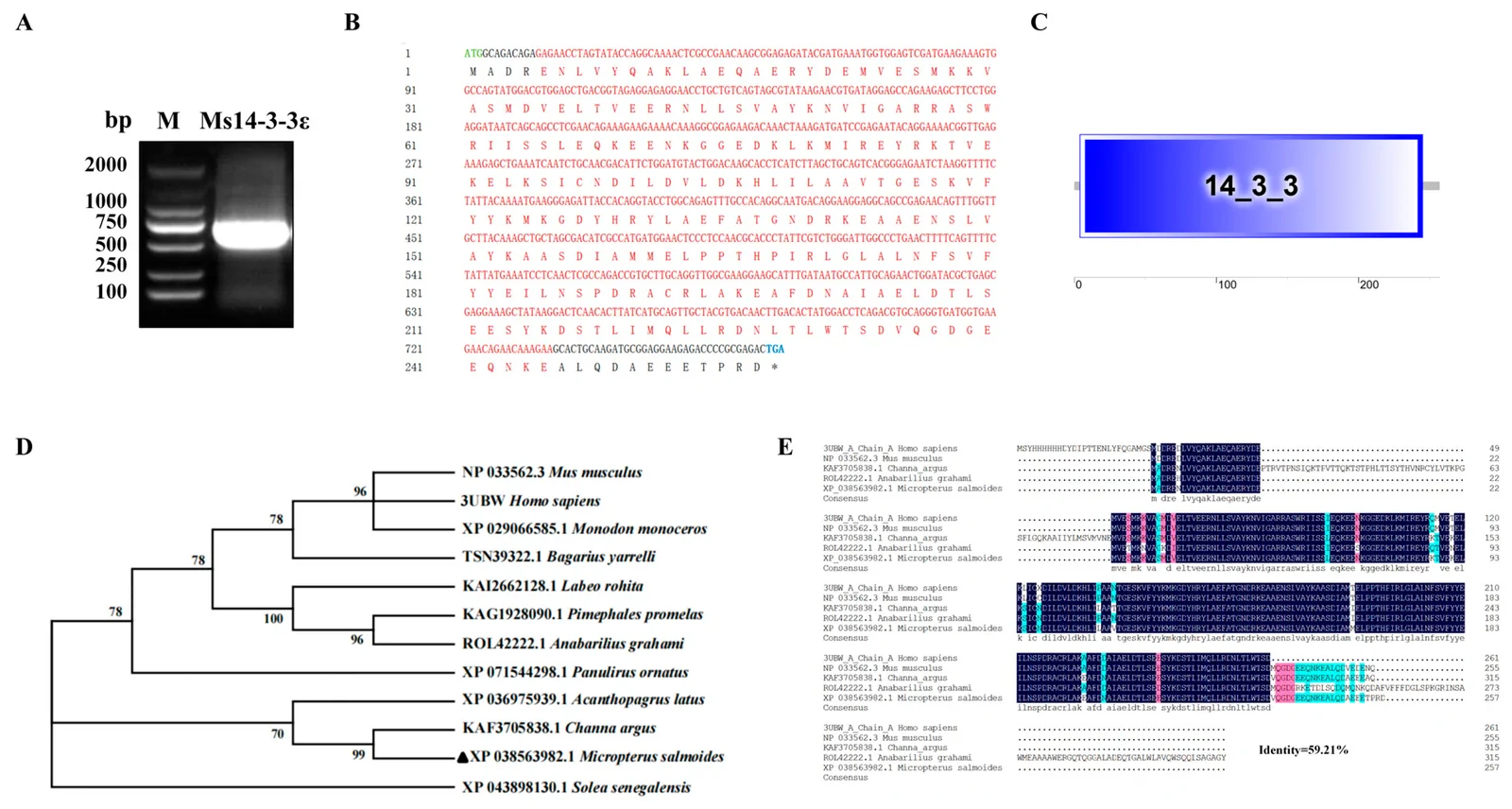
Molecular cloning and sequence characterization of *Ms14-3-3ε*. (**A**) PCR amplification of the full-length ORF of Ms14-3-3ε. M: DNA marker. (**B**) The nucleotide and deduced amino acid sequence of *Ms14-3-3ε*. The start codon (ATG) is labeled in green, the termination codon (TGA) is marked with an asterisk (*) and labeled in blue, and the conserved 14-3-3 functional domain is highlighted in red. (**C**) The putative functional domain of *Ms14-3-3ε*. The domain structure was predicted using the SMART database. The 14-3-3 domain was highlighted in blue; the numbers indicated the amino acid positions. (**D**) Phylogenetic tree analysis of Ms14-3-3ε. A neighbor-joining (NJ) phylogenetic tree was constructed based on full-length amino acid sequences of the indicated 14-3-3ε proteins. The numbers marked on the tree branches represent the bootstrap values. The location of Ms14-3-3ε is indicated by a triangle. (**E**) Multiple sequence alignment analysis of Ms14-3-3ε and its homologous 14-3-3ε proteins from different species. The following 14-3-3εs were used: largemouth bass (*M. salmoides*) 14-3-3ε (XP_038563982.1), *Homo sapiens* 14-3-3ε (Hm14-3-3ε, 3UBW_A), *Anabarilius grahami* 14-3-3ε (Ag14-3-3ε, ROL42222.1), *Mus musculus* 14-3-3ε (Mm14-3-3ε, NP_033562.3) and *Channa argus* 14-3-3ε (Ca14-3-3ε, KAF3705838.1). Overall sequence identity among these proteins was shown at the end.

The Ms14-3-3ε ORF comprises 774 bp, encoding a predicted protein of 257 amino acids with an estimated molecular weight of 29.39 kDa (Figure 1A,B). Conserved domain architecture analysis indicated that Ms14-3-3ε contains a highly conserved, canonical 14-3-3 domain spanning amino acids 4 to 245 (Figure 1C). To explore its evolutionary relationship among vertebrates, a neighbor-joining phylogenetic tree was constructed. Ms14-3-3ε clustered tightly into the teleost 14-3-3ε clade, sharing the highest homology with *Channa argus* (Figure 1D). Multiple sequence alignment further demonstrated that the amino acid sequence of Ms14-3-3ε shares 59.21% global identity with 14-3-3ε orthologs from other species, with core functional domains showing exceptionally high site-specific conservation compared to peripheral variable regions (Figure 1E).

### 3.2. Tissue Distribution and Transcriptional Responses of Ms14-3-3ε to Viral Infection

**Figure 2:**
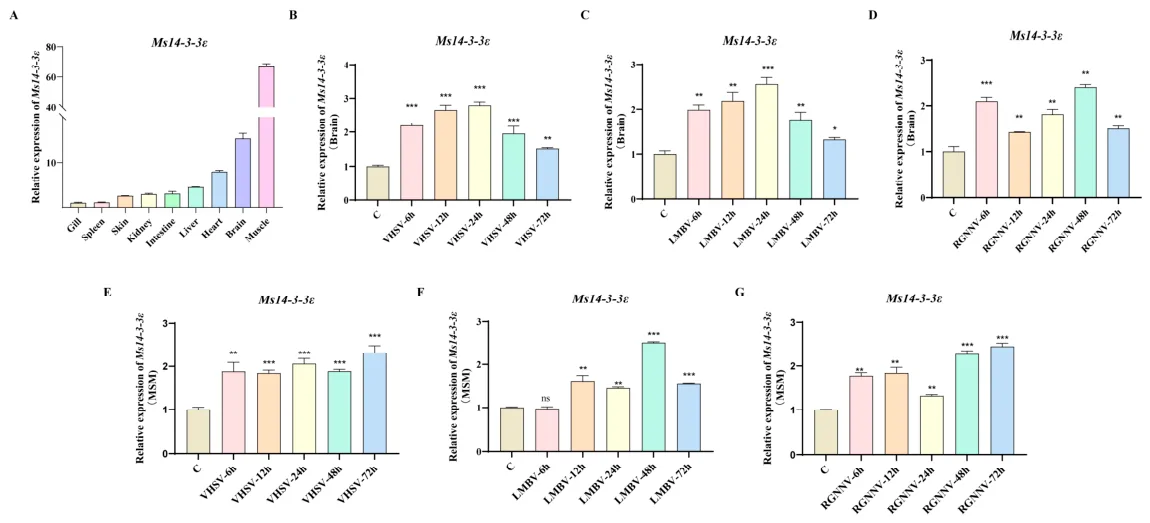
Tissue distribution and virus-induced expression profiles of *Ms14-3-3ε* in vivo and in vitro. (**A**) Basal tissue distribution of *Ms14-3-3ε* transcripts across multiple healthy largemouth bass (*M. salmoides*) tissues. Tissues were collected from nine healthy fish; tissues from three fish were pooled as one biological replicate (*n* = 3 per tissue). (**B**–**D**) Expression level of *Ms14-3-3ε* in brain tissues of largemouth bass (*M. salmoides*) infected with VHSV, LMBV and RGNNV at 0, 6, 12, 24, 48, and 72 h post-infection (hpi). (**E**–**G**) Relative expression levels of *Ms14-3-3ε* in MSM cells after VHSV, LMBV, and RGNNV infection at 0, 6, 12, 24, 48, and 72 h. In panels B-G, “C” indicates the PBS control sampled at 0 hpi; infected groups were compared against this baseline. One-way ANOVA with Dunnett’s multiple-comparison test; asterisks indicate comparison of each time point versus 0 hpi. Data are mean ± SD; *n* = 3 biological replicates. ns, not significant; *, *p* < 0.05; **, *p* < 0.01; ***, *p* < 0.001.

*Ms14-3-3ε* mRNA was ubiquitously detected across all examined tissues, including the gill, spleen, skin, kidney, intestine, liver, brain, heart, and muscle, with the highest expression observed in muscle and the lowest in the gill (Figure 2A). To determine whether Ms14-3-3ε responds to viral challenges, its transcription dynamics were monitored following in vivo injection or in vitro incubation with VHSV, LMBV, and RGNNV. Notably, *Ms14-3-3ε* mRNA levels in both largemouth bass brain tissues (Figure 2B–D) and MSM cells (Figure 2E–G) were significantly upregulated to varying degrees post-infection. These findings suggest that Ms14-3-3ε actively participates in the host response to VHSV, LMBV, and RGNNV infections.

### 3.3. Ms14-3-3ε Promotes VHSV, LMBV and RGNNV Infections

**Figure 3:**
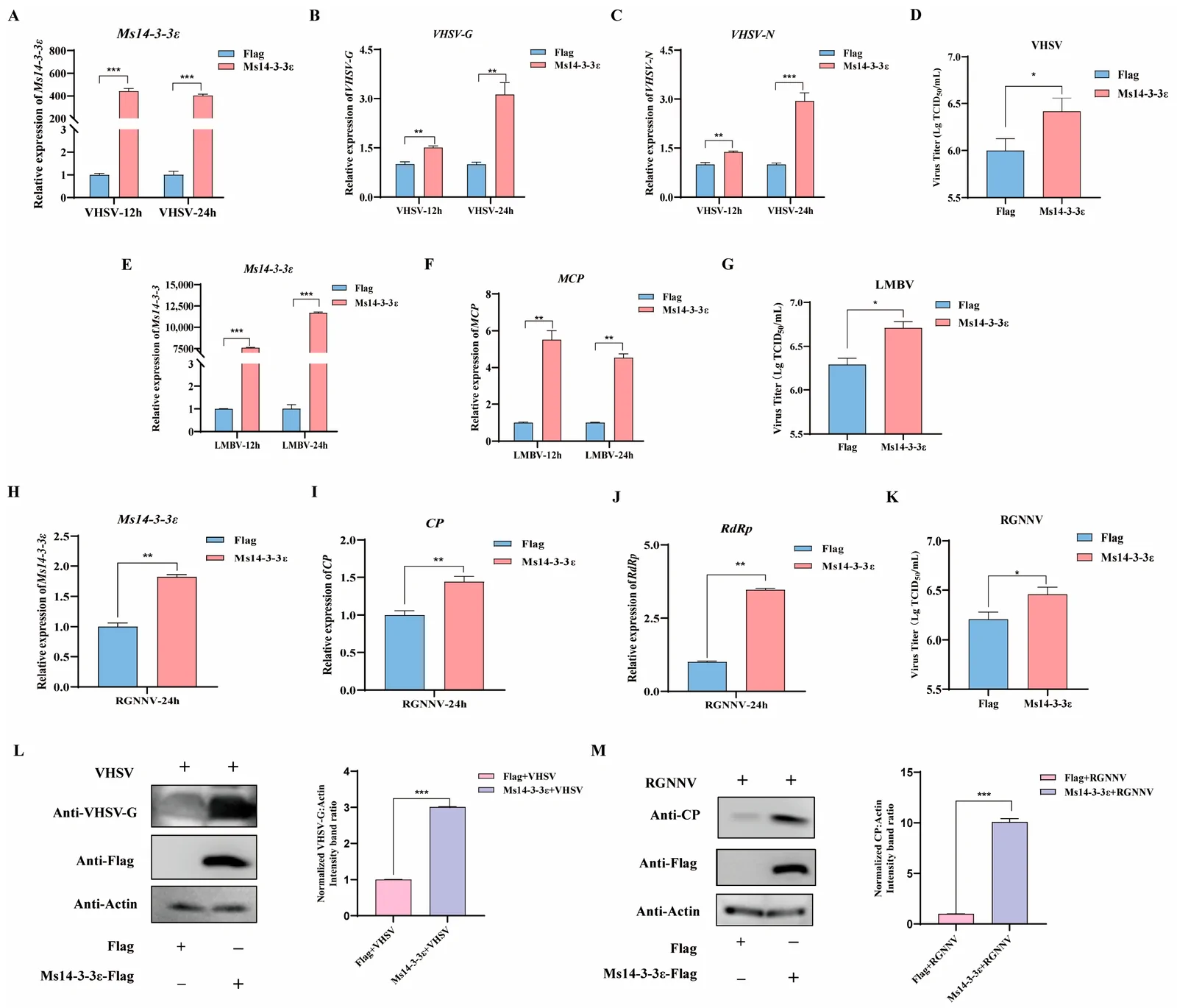
Effect of Ms14-3-3ε overexpression on viral replication in MSM cells. (**A**–**C**) mRNA expression levels of *Ms14-3-3ε* (**A**), *VHSV G* (**B**), and VHSV *N* (**C**) at 12 and 24 hpi following VHSV infection after *Ms14-3-3ε* overexpression. (**D**) Effect of *Ms14-3-3ε* overexpression on VHSV infection titer. MSM cells were transfected with pCMV-Flag-*Ms14-3-3ε* or the empty vector pCMV-Flag for 24 h, followed by infection with VHSV. Culture supernatants were collected at 24 hpi, and infectious viral titers were determined by TCID_50_ assay. (**E**,**F**) Expression levels of *Ms14-3-3ε* (**E**) and *MCP* (**F**) at 12 and 24 hpi following LMBV infection after *Ms14-3-3ε* overexpression. (**G**) *Ms14-3-3ε* overexpression increases LMBV titer. MSM cells transfected with pCMV-Flag-*Ms14-3-3ε* or pCMV-Flag were infected with LMBV. Culture supernatants were collected at 24 hpi, and viral titers were determined by TCID_50_ assay. (**H**–**J**) qRT-PCR analysis of *Ms14-3-3ε* (**H**)*, CP* (**I**) and *RdRp* (**J**) at 24 hpi in *Ms14-3-3ε*-overexpressing MSM cells. (**K**) RGNNV titer in *Ms14-3-3ε*-overexpressing MSM cells. Cells were infected with RGNNV, and supernatants were collected at 24 hpi for TCID_50_ assay. (**L**) VHSV G protein expression in Ms14-3-3ε-overexpressed MSM cells was analyzed by Western blotting at 24 hpi. (**M**) CP protein expression in Ms14-3-3ε-overexpressed MSM cells was analyzed by Western blotting at 24 hpi. Data are presented as mean ± SD (*n* = 3 biological replicates). For Figure 3A–C,E,F, two-way ANOVA with treatment and time as factors followed by Šídák’s multiple-comparison test was used. For Figure 3D,G,H–M, a two-tailed Student’s *t*-test was used. *, *p* < 0.05; **, *p* < 0.01; ***, *p* < 0.001.

**Figure 4:**
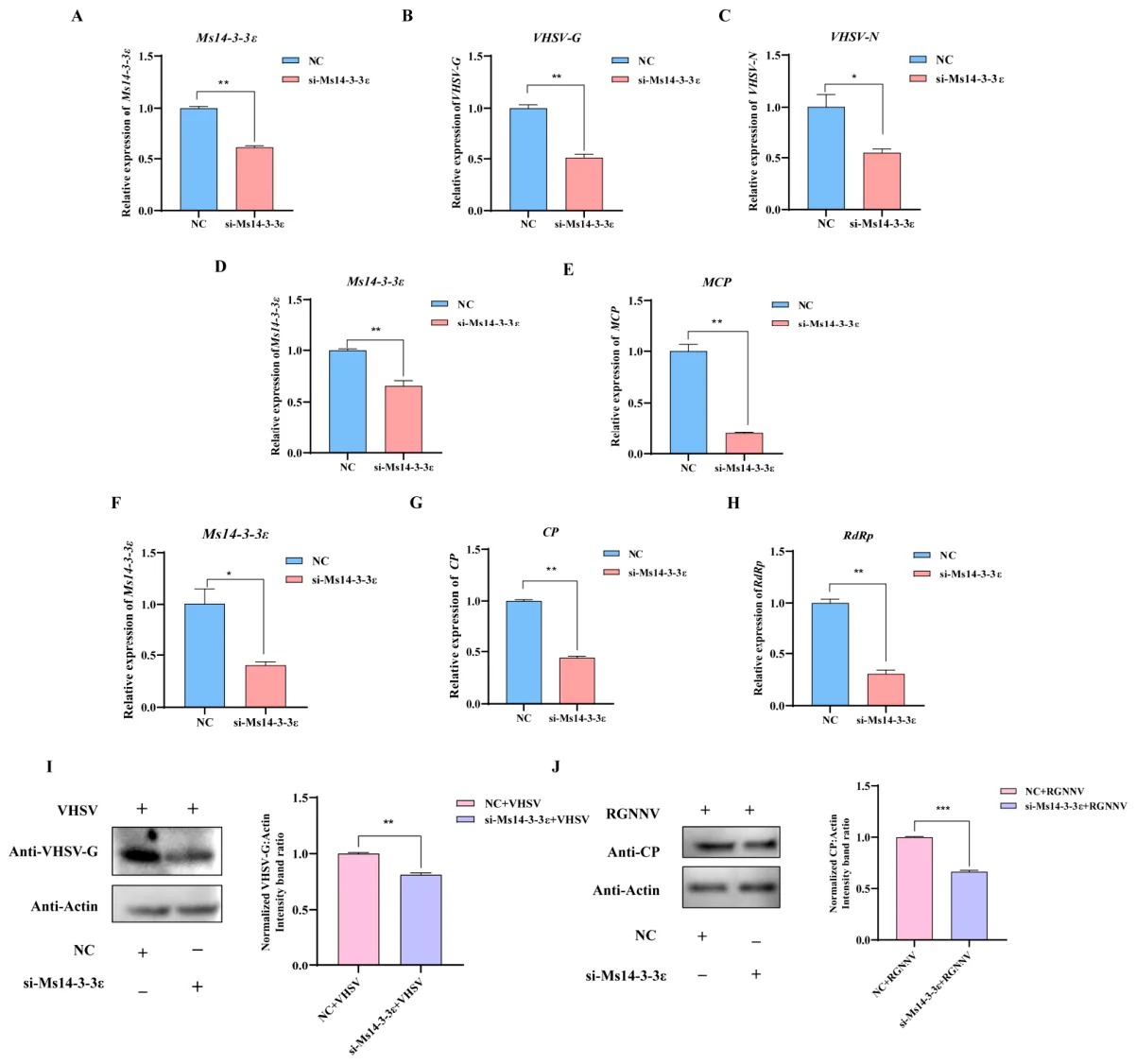
Knockdown of Ms14-3-3ε suppresses the expression of VHSV, LMBV, and RGNNV viral genes. (**A**–**C**) Upon *Ms14-3-3ε* knockdown, mRNA levels of *Ms14-3-3ε* (**A**), *VHSV G* (**B**) and *VHSV N* (**C**) were measured at 24 hpi following VHSV infection. (**D**,**E**) Relative expression of *Ms14-3-3ε* (**D**) and *MCP* (**E**) at 24 hpi in Ms14-3-3ε-knockdown cells after LMBV challenge. (**F**–**H**) qRT-PCR detection of *Ms14-3-3ε* (**F**), *CP* (**G**) and *RdRp* (**H**) expression at 24 hpi in Ms14-3-3ε-knockdown MSM cells. (**I**) VHSV G protein expression was analyzed by Western blotting at 24 hpi in Ms14-3-3ε-knockdown MSM cells. (**J**) CP protein levels were evaluated by Western blotting at 24 hpi in Ms14-3-3ε-knockdown MSM cells following RGNNV infection. Data are presented as mean ± SD. Student’s t-test was used to determine significance (*, *p* < 0.05; **, *p* < 0.01; ***, *p* < 0.001).

To explore the biological function of Ms14-3-3ε during viral infection, loss- and gain-of-function strategies were deployed in MSM cells. MSM cells were transfected with pCMV-Flag-Ms14-3-3ε for 24 h and subsequently challenged with VHSV, LMBV, or RGNNV. The results showed that overexpression of Ms14-3-3ε markedly upregulated the mRNA levels of essential viral genes, including *VHSV-N*, *VHSV-G*, *LMBV-MCP*, *RGNNV-CP*, and *RGNNV-RdRp* (Figure 3A–C,E,F,H–J). TCID_50_ assays showed that Ms14-3-3ε overexpression significantly increased the infectious titers of VHSV, LMBV, and RGNNV, consistent with the viral RNA results (Figure 3D,G,K). Further Western blot analysis verified that Ms14-3-3ε overexpression markedly increased the protein expression levels of VHSV G and RGNNV CP (Figure 3L–M). Conversely, silencing of Ms14-3-3ε via specific siRNA led to a dramatic reduction in the transcript levels of these viral genes compared with the negative control group (Figure 4A–H). Consistently, the protein abundances of VHSV-G and RGNNV-CP were concurrently suppressed in Ms14-3-3ε-knockdown cells (Figure 4I–J). Taken together, these results demonstrate that Ms14-3-3ε acts as a broad-spectrum pro-viral factor that promotes the replication of VHSV, LMBV, and RGNNV.

### 3.4. Ms14-3-3ε Negatively Regulates the NF-κB Signaling Pathway

**Figure 5:**
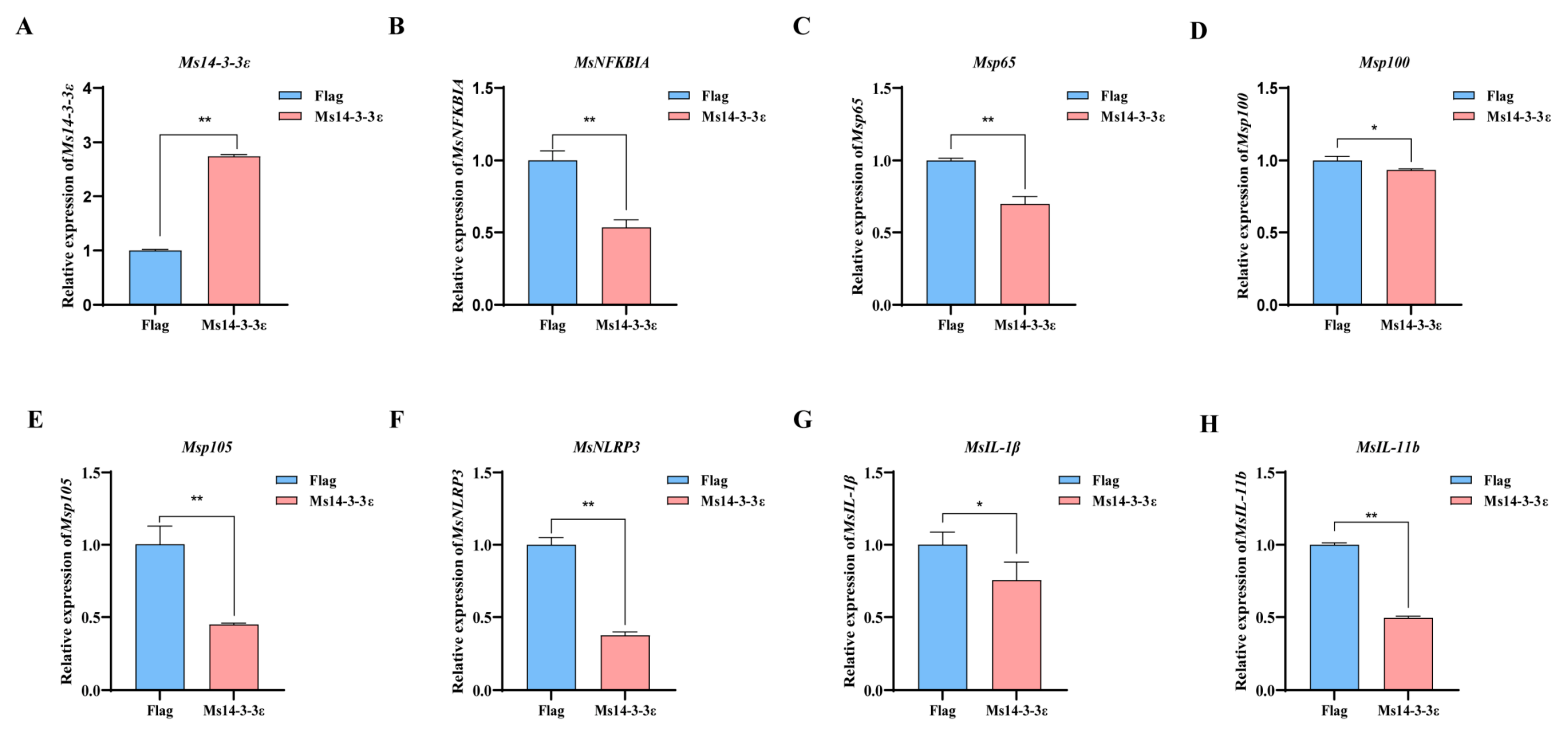
Ms14-3-3ε overexpression suppresses the expression of NF-κB-related genes. (**A**–**H**) qRT-PCR analysis of *Ms14-3-3ε* (**A**), *MsNFKBIA* (**B**), *Msp65* (**C**), *Msp100* (**D**), *Msp105* (**E**), *MsNLRP3* (**F**), *MsIL-1β* (**G**), and *MsIL-11b* (**H**) in Ms14-3-3ε-overexpressed MSM cells. *Msβ-actin* served as an internal reference for qRT-PCR. All the data were provided as the Mean ± SD of at least three assays and analyzed statistically by Student’s *t*-test (*, *p* < 0.05; **, *p* < 0.01).

**Figure 6:**
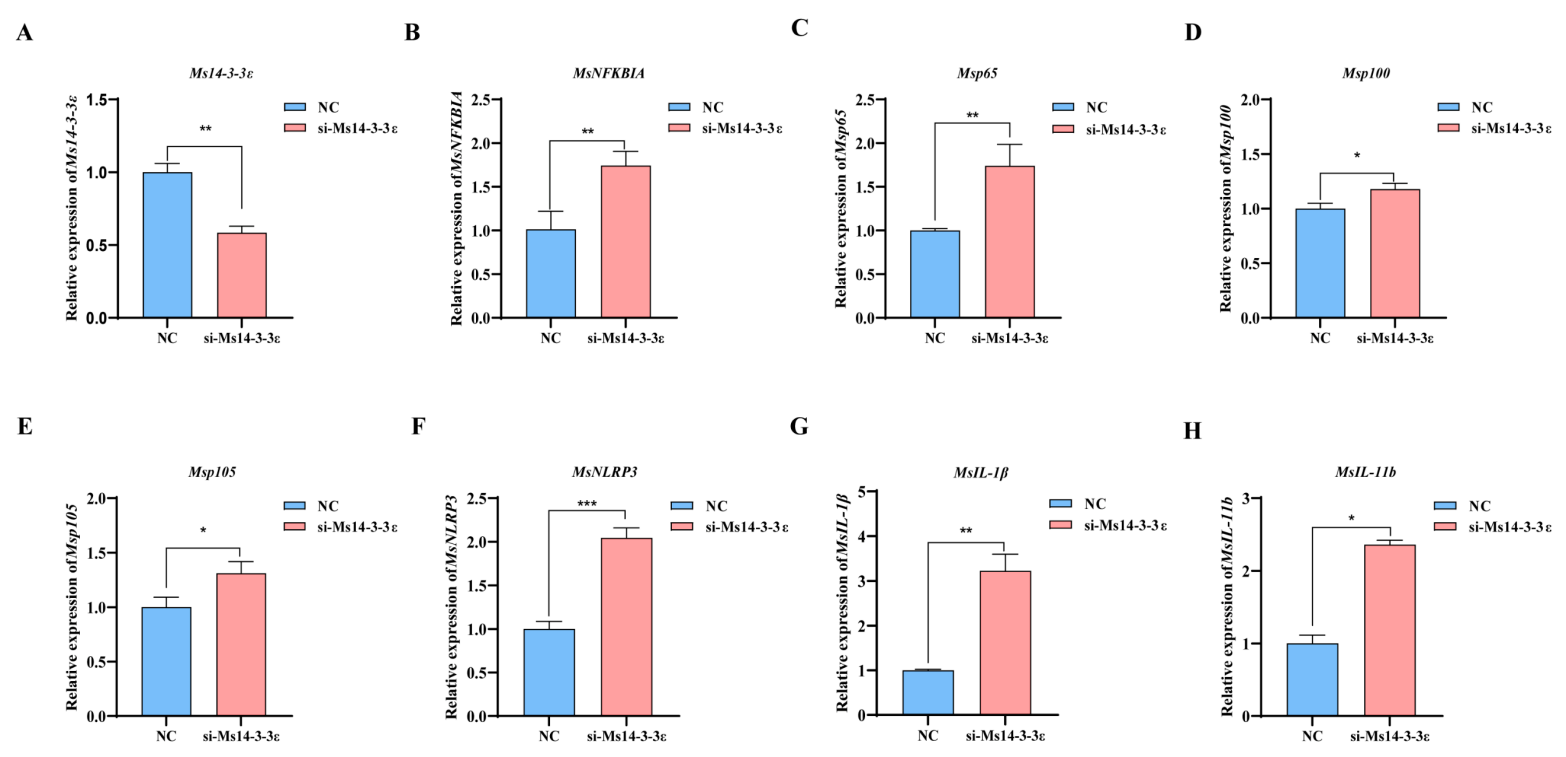
Knockdown of Ms14-3-3ε facilitates the activation of NF-κB-mediated inflammatory genes. MSM cells were transfected with si-Ms14-3-3ε (NC as control) for 24 h. The mRNA levels of *Ms14-3-3ε* (**A**), *MsNFKBIA* (**B**), *Msp65* (**C**), *Msp100* (**D**), *Msp105* (**E**), *MsNLRP3* (**F**), *MsIL-1β* (**G**), and *MsIL-11b* (**H**) were then determined by qRT-PCR. *Msβ-actin* was used as an internal reference. Data are presented as mean ± SD from at least three independent assays, and statistical significance was analyzed by Student’s *t*-test (*, *p* < 0.05; **, *p* < 0.01; ***, *p* < 0.001).

Given that mammalian 14-3-3ε can negatively modulate the NF-κB pathway [28], we investigated whether this regulatory mechanism is conserved in teleosts to explain how Ms14-3-3ε facilitates viral replication. The mRNA levels of key components and downstream effector molecules of the NF-κB signaling pathway were detected following the overexpression and knockdown of *Ms14-3-3ε*, respectively. As shown in Figure 5, overexpression of *Ms14-3-3ε* significantly downregulated the mRNA levels of *MsNFKBIA*, *Msp65*, *Msp100*, and *Msp105*, as well as the downstream pro-inflammatory factors *MsNLRP3*, *MsIL-1β,* and *MsIL-11b* in MSM cells. Conversely, knockdown of *Ms14-3-3ε* via siRNA led to a marked increase in the transcription of these genes (Figure 6A–H). Taken together, these data demonstrate that Ms14-3-3ε acts as a negative regulator of the NF-κB signaling pathway in largemouth bass (*M. salmoides*).

### 3.5. Ms14-3-3ε Inhibits Virus-Induced Activation of the NF-κB Promoter

**Figure 7:**
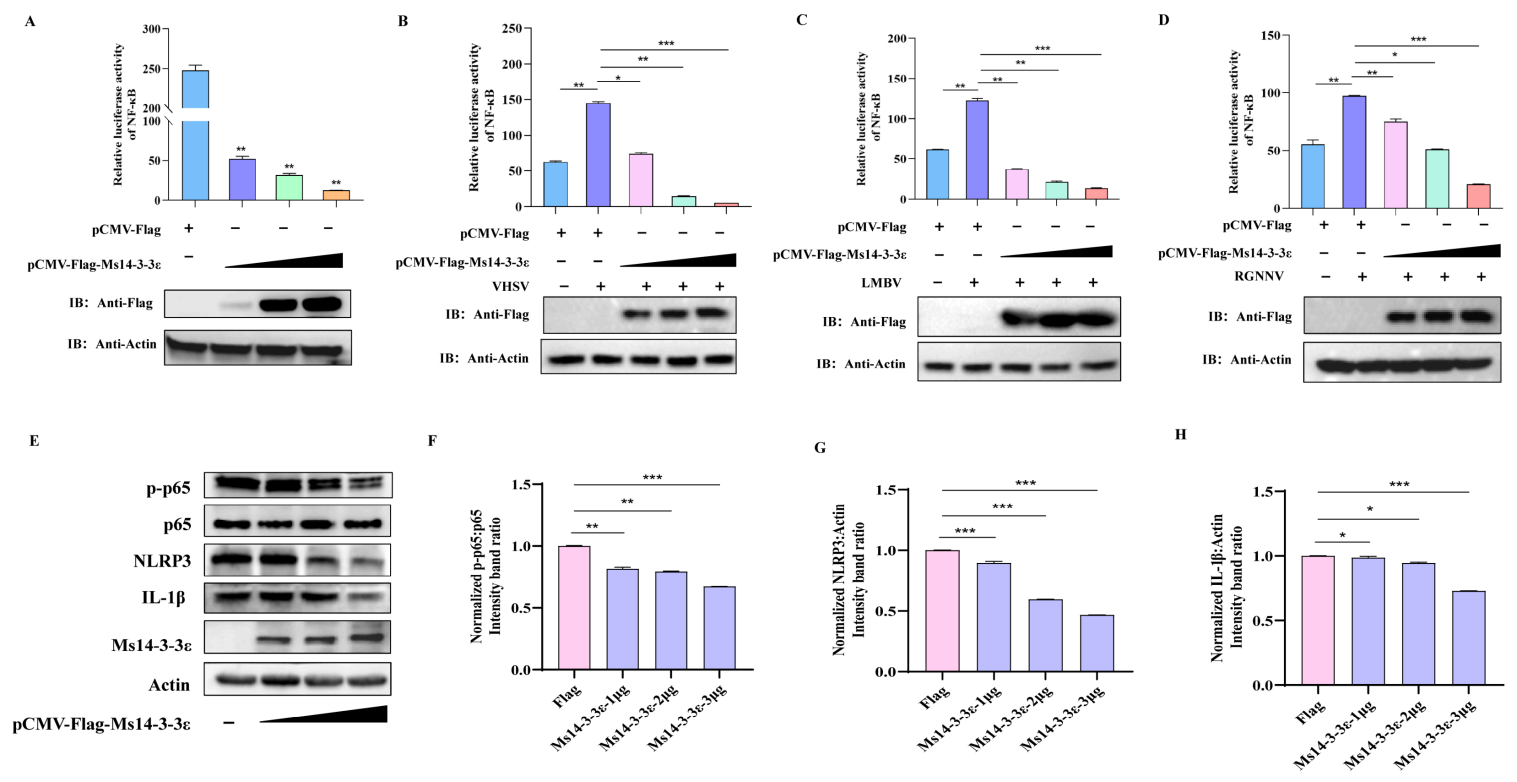
Ms14-3-3ε negatively modulates NF-κB signaling activation in MSM cells. (**A**) MSM cells were co-transfected with 0.25, 0.5, and 0.75 μg of pCMV-Flag-Ms14-3-3ε, pNF-κB-Luc, and pRL-TK plasmids and an empty vector was added to equalize the total amount of plasmids across all groups. At 24 h post-transfection, cells were harvested and lysed for a dual-luciferase reporter assay (*n* = 3). (**B**–**D**) MSM cells were co-transfected with pNF-κB-Luc and pRL-TK together with pCMV-Flag or 0.25, 0.5, and 0.75 μg of pCMV-Flag-Ms14-3-3ε. At 24 h post-transfection, cells were treated with PBS or infected with VHSV (**B**), LMBV (**C**), or RGNNV (**D**). Cells were harvested and lysed for a dual-luciferase reporter assay at 24 h post-infection. (**E**) Western blot analysis of Ms14-3-3ε-Flag, β-actin, phosphorylated p65 (p-p65), total p65, NLRP3, and IL-1β in MSM cells transfected with 0, 1, 2, and 3 μg of pCMV-Flag-Ms14-3-3ε. pCMV-Flag vector was used to equalize the total amount of transfected plasmid. (**F**) Quantitative densitometric analysis of p-p65 protein levels normalized to p65. (**G**,**H**) NLRP3 (**G**) and IL-1β (**H**) protein levels normalized to β-actin. Data are mean ± SD (*n* = 3 biological replicates). One-way ANOVA followed by Dunnett’s test versus the 0 μg group was used for Figure 7A,F–H; two-way ANOVA with virus infection and plasmid dose as factors followed by Tukey’s test was used for Figure 7B–D. *, *p* < 0.05; **, *p* < 0.01; ***, *p* < 0.001.

To further validate the negative regulation of Ms14-3-3ε on NF-κB signaling at the transcriptional level, a dual-luciferase reporter assay was applied to examine the effect of Ms14-3-3ε on NF-κB transcriptional activity. In MSM cells, transfection of *pCMV-Flag-Ms14-3-3ε* suppressed the basal activity of the NF-κB promoter in a dose-dependent manner (Figure 7A). Furthermore, while challenges with VHSV, LMBV, and RGNNV robustly activated the NF-κB promoter, co-transfection with increasing amounts of *pCMV-Flag-Ms14-3-3ε* effectively neutralized virus-induced promoter activation in a dose-dependent fashion (Figure 7B–D). These data indicate that Ms14-3-3ε suppresses not only the basal activity of the NF-κB promoter but also virus-triggered NF-κB activation.

### 3.6. Ms14-3-3ε Suppresses the Phosphorylation of p65 and Downstream Inflammatory Effectors

To further decipher the protein-level dynamics of this regulatory mechanism, immunoblotting was performed following transfection with increasing doses of pCMV-Flag-Ms14-3-3ε. While the total protein level of the core transcription factor p65 remained unchanged, the abundance of phosphorylated p65 (p-p65 at Ser536) exhibited a pronounced, dose-dependent decline (Figure 7E,F). Concurrently, the protein expression of the downstream inflammatory effectors NLRP3 and IL-1β was significantly reduced with increasing concentrations of Ms14-3-3ε (Figure 7E,G,H). These findings show that, under the tested conditions, total p65 levels were unchanged while its phosphorylation at Ser536 was reduced in a dose-dependent manner; the abundance of the downstream proteins NLRP3 and IL-1β was concurrently reduced.

## 4. Discussion

Frequent outbreaks of mixed viral infections or heterologous polyviral co-infections represent a critical bottleneck hindering the sustainable aquaculture of economically important teleosts. Because traditional single-target prevention and control strategies are largely ineffective against multi-pathogen complexes, identifying conserved host factors leveraged by diverse viruses is of profound scientific and practical value [29]. In this study, the Ms14-3-3ε was successfully cloned and functionally characterized. Mechanistically, Ms14-3-3ε facilitates infection by three distinct viruses belonging to different nucleic acid lineages (VHSV, LMBV, and RGNNV) by negatively regulating the NF-κB signaling pathway. These findings identify Ms14-3-3ε as a broad-spectrum pro-viral host factor in largemouth bass, offering a promising target for developing pan-viral countermeasures in aquaculture. Notably, although the three viruses were tested individually, this design allowed a stringent assessment of whether a single host factor is convergently exploited by phylogenetically distinct pathogens and provides a baseline for future studies under co-infection conditions.

Structurally, 14-3-3 monomers fold into a canonical spatial architecture of nine antiparallel α-helices, forming a homo- or heterodimeric groove that binds to phosphorylated serine/threonine (pS/pT) motifs on target proteins to act as central decoders of cellular phosphorylation signaling [30]. Sequence analysis revealed that Ms14-3-3ε consists of 257 amino acids and possesses a typical, highly conserved 14-3-3 domain. Critical residues involved in ligand binding and dimerization interfaces are strictly conserved, sharing over 50% identity with mammalian 14-3-3ε orthologs [31]. This deep sequence preservation suggests that 14-3-3ε has been under stringent purifying evolutionary constraints, highlighting its fundamental biological importance in cellular regulation. Tissue distribution analysis showed that Ms14-3-3ε is ubiquitously expressed across all examined tissues in healthy fish, which matches its role as a cellular “housekeeping protein” required for basal homeostatic maintenance, consistent with reports in other teleosts like rainbow trout [21]. Remarkably, *Ms14-3-3ε* mRNA expression was significantly upregulated in largemouth bass brain tissues and MSM cells upon infection with VHSV, LMBV and RGNNV. This broad transcriptional stress response mirrors the upregulation of 14-3-3ε in *Litopenaeus vannamei* challenged with WSSV [32] and in human A549 cells infected with influenza A virus (IAV) [33]. The structural conservation and universal response of 14-3-3ε across divergent host–virus systems emphasize its role as a vital hub in virus–host interactions.

The regulatory roles of 14-3-3ε during viral infections are complex and context-dependent. While 14-3-3ε can act as an immune accelerator by bridging RIG-I and TRIM25 to trigger MAVS-mediated antiviral defense against certain RNA viruses [13], many pathogens have evolved sophisticated mechanisms to hijack or disrupt 14-3-3ε to suppress host defenses. For instance, the IAV NS1 protein directly interacts with 14-3-3ε to block RIG-I mitochondrial translocation [34], while coxsackievirus B3 utilizes its 3C protease to cleave 14-3-3ε into dominant-negative fragments that cannot bind RIG-I [35]. Thus, 14-3-3ε acts both as an accelerator of innate immune activation and as a vulnerable target exploited by viruses to dampen host defense. In the present study, overexpressing Ms14-3-3ε markedly enhanced the replication of VHSV, LMBV, and RGNNV, indicating that Ms14-3-3ε functions as a pro-viral host factor that increases largemouth bass susceptibility to these pathogens. This phenotype is consistent with observations in mammals, where 14-3-3ε is commonly hijacked by diverse viruses to evade immune clearance and support viral propagation, suggesting possible functional conservation of vertebrate 14-3-3ε in mediating host susceptibility to heterologous viral pathogens. While previous mammalian studies have predominantly focused on the relationship between 14-3-3ε and a single RNA virus [11], our findings reveal that Ms14-3-3ε not only promotes the replication of the RNA viruses VHSV and RGNNV but also dramatically enhances the propagation of the double-stranded DNA virus LMBV. This pro-viral effect across the three viruses tested suggests that Ms14-3-3ε may exert its effects via conserved host signaling pathways; however, the specific mechanism and whether it interacts directly with viral or host factors remain to be further investigated.

To resolve the broad-spectrum mechanism by which Ms14-3-3ε facilitates the replication of these three distinct viruses, we examined the NF-κB pathway, a central axis governing innate immunity and inflammation in teleosts [36,37]. Upon activation, NF-κB triggers the nuclear translocation of p65 to drive the transcription of antiviral effectors and pro-inflammatory mediators, including the cytokine IL-1β and the inflammasome-associated protein NLRP3, which serve as crucial lines of defense against aquatic pathogens [38]. Accumulating evidence indicates that the 14-3-3 protein family is an important modulator of NF-κB signaling, and numerous viruses have evolved classic immune evasion strategies to hijack 14-3-3 proteins and inhibit NF-κB activation. For instance, Nipah virus W protein sequesters 14-3-3 in the nucleus to suppress p65 Ser536 phosphorylation and nuclear translocation, thereby inhibiting proinflammatory signaling [39]. Similarly, vaccinia virus virulence factor B14 binds IKKβ to prevent upstream NF-κB activation [40], whereas KSHV miR-K1 targets IκBα to reduce IκBα protein levels [41], thereby activating NF-κB to maintain viral latency. Here, Ms14-3-3ε was shown to exert a potent inhibitory effect on the NF-κB pathway in largemouth bass. Overexpression of Ms14-3-3ε significantly blunted the NF-κB activation triggered by VHSV, LMBV, and RGNNV infections. Crucially, while total p65 protein levels remained unaltered under the tested conditions, the phosphorylation of p65 at Ser536 was suppressed in a dose-dependent manner, demonstrating that Ms14-3-3ε may block the transcriptional activation of p65 by interfering with its phosphorylation modification. In parallel, the protein levels of the NF-κB downstream molecules NLRP3 and IL-1β (precursor form, ~31 kDa) were also markedly downregulated. Because the NLRP3 inflammasome complex is a principal sensor governing IL-1β maturation and secretion [42], these reductions may reflect decreased NF-κB pathway activity under the tested conditions. Collectively, these findings suggest that Ms14-3-3ε may suppress the host antiviral innate immune response by targeting the NF-κB axis, generating an immunosuppressive intracellular microenvironment that potentially facilitates the replication of VHSV, LMBV, and RGNNV.

It should be noted that the present study has several limitations. First, although Ms14-3-3ε was shown to inhibit p65 phosphorylation and NF-κB activation, the pro-viral phenotype may additionally involve direct interactions with other host factors in the viral replication cycle or signaling pathways other than NF-κB, given the promiscuous phospho-binding nature of 14-3-3 proteins; rescue experiments (e.g., p65 activation or NF-κB inhibition following Ms14-3-3ε silencing) and interaction assays between Ms14-3-3ε and p65 or its upstream kinase will be required to establish causality. Second, all functional experiments were performed in single-virus models. Therefore, the present conclusions are limited to VHSV, LMBV, and RGNNV in the MSM cell model, and cannot be directly extrapolated to co-infection settings. Whether Ms14-3-3ε retains this broad-spectrum pro-viral function under natural co-infection conditions warrants future investigation.

## 5. Conclusions

In summary, this study identifies Ms14-3-3ε as a pro-viral host factor for VHSV, LMBV, and RGNNV in the MSM cell model. Ms14-3-3ε enhances the replication of these three viruses, accompanied by reduced p65 phosphorylation and attenuated NF-κB pathway activity, including reduced levels of the downstream inflammasome-associated protein NLRP3 and the cytokine IL-1β. These findings provide a novel host target for developing broad-spectrum antiviral strategies against fish pathogens.

## Data Availability

Data will be made available on request.

## Supplementary Materials

The following supporting information can be downloaded at: https://www.mdpi.com/article/10.3390/ani16213368/s1, Table S1: Sequences of primers and siRNAs used in this study.
