# Supplementary material for: 14-3-3ε Facilitates Replication of Multiple Viruses Accompanied by Reduced NF-κB Pathway Activity in Largemouth Bass (Micropterus salmoides)
Source: Animals (Basel). 2026 Oct 25;16(21):3368. doi: 10.3390/ani16213368 (PMC13761646; doi:10.3390/ani16213368)
Supplement: Supplementary file 1 [file animals-16-03368-s001.zip › animals-4568438-supplementary.pdf]

Table S1

Sequences of primers and siRNAs used in this study.

Name	Sequence (5′-3′)
**For gene cloning**	
Ms14-3-3ε-F	CCATGGAGGCCCGAATTCGGATGGCAGACAGAGAGAACCT
Ms14-3-3ε-R	ATCCCCGCGGCCGCGGTACCTCAGTCTCGCGGGGTCTCTT
**For qPCR analysis**	
Ms14-3-3ε-Q-F	ACAGGCAATGACAGGAAGGAG
Ms14-3-3ε-Q-R	AAGCACGGTCTGGCGAGTT
MsNFKBIA-Q-F	AACAACCTCCCGCTGTAACC
MsNFKBIA-Q-R	CGGAGTCTGTCTCTGGTCGT
Msp65-Q-F	AAGCAGAGGGGGATGAGGTT
Msp65-Q-R	TTCGTCACCAATGAGATGCG
Msp100-Q-F	GGACATAAAGAGCGAACCCTACA
Msp100-Q-R	TTCTCCTGTTCTTCTCGCTGG
Msp105-Q-F	AAACGCCAATGTGGACTGCT
Msp105-Q-R	ATCTTCATCCTCATCCTCCCTCT
MsNLRP3-Q-F	CAGCCTGGAGAACACCAAGT
MsNLRP3-Q-R	GGTCTCCTCCTTGTTGCTGA
MsIL-1β-Q-F	CGACCGCAGTAAGAAAGACATC
MsIL-1β-Q-R	GATGGACAGAACAACGGGACTA
MsIL-11b-Q-F	TTTGTCCTCCCACTCGGCT
MsIL-11b-Q-R	GAACTGGAGCACATCAAAGGC
Msβ-actin-Q-F	CAGACTACCTCATGAAGATCCT
Msβ-actin-Q-R	CTGTCCGTCAGGCAGCTCG
VHSV-G-Q-F	AAAGAACTGAGTGCCTGGACG
VHSV-G-Q-R	CTCGTAATCACACTCACCTCGC
VHSV-N-Q-F	TTGGAGAACTGCAACACTTCAC
VHSV-N-Q-R	CGGTCAGGATGAAGGCGTAG
MCP-Q-F	CCAAAAATCTCATGCACAACG
MCP-Q-R	GGGGCGGGATTGACGAGATC
CP-Q-F	GTCGGCTGATACTCCTGTGTG

CP-Q-R	CTCCAGTTCCAAGGCTGTAGT
RdRp-Q-F	GCTTTATGCGTGAGTGCGTC
RdRp-Q-R	GCTGTTTCCGTCTGTTGTGAG
**For siRNA analysis**	
si-Ms14-3-3ε-1-sense	GGCGGAGAAGACAAACUAA(dT)(dT)
si-Ms14-3-3ε-1-antisense	UUAGUUUGUCUUCUCCGCC(dT)(dT)
si-Ms14-3-3ε-2-sense	GGACAAGCACCUCAUCUUA(dT)(dT)
si-Ms14-3-3ε-2-antisense	UAAGAUGAGGUGCUUGUCC(dT)(dT)
si-Ms14-3-3ε-3-sense	GCGGAGAGAUACGAUGAAA(dT)(dT)
si-Ms14-3-3ε-3-antisense	UUUCAUCGUAUCUCUCCGC(dT)(dT)
